# Mitigation of infectious disease at school: targeted class closure vs school closure

**DOI:** 10.1186/s12879-014-0695-9

**Published:** 2014-12-31

**Authors:** Valerio Gemmetto, Alain Barrat, Ciro Cattuto

**Affiliations:** Data Science Laboratory, ISI Foundation, Turin, Italy; Aix Marseille Université, Université de Toulon, CNRS, CPT, UMR 7332, Marseille, 13288 France

**Keywords:** Infectious diseases, School contact networks, Targeted interventions

## Abstract

**Background:**

School environments are thought to play an important role in the community spread of infectious diseases such as influenza because of the high mixing rates of school children. The closure of schools has therefore been proposed as an efficient mitigation strategy. Such measures come however with high associated social and economic costs, making alternative, less disruptive interventions highly desirable. The recent availability of high-resolution contact network data from school environments provides an opportunity to design models of micro-interventions and compare the outcomes of alternative mitigation measures.

**Methods and results:**

We model mitigation measures that involve the targeted closure of school classes or grades based on readily available information such as the number of symptomatic infectious children in a class. We focus on the specific case of a primary school for which we have high-resolution data on the close-range interactions of children and teachers. We simulate the spread of an influenza-like illness in this population by using an SEIR model with asymptomatics, and compare the outcomes of different mitigation strategies. We find that targeted class closure affords strong mitigation effects: closing a class for a fixed period of time – equal to the sum of the average infectious and latent durations – whenever two infectious individuals are detected in that class decreases the attack rate by almost 70% and significantly decreases the probability of a severe outbreak. The closure of all classes of the same grade mitigates the spread almost as much as closing the whole school.

**Conclusions:**

Our model of targeted class closure strategies based on readily available information on symptomatic subjects and on limited information on mixing patterns, such as the grade structure of the school, shows that these strategies might be almost as effective as whole-school closure, at a much lower cost. This may inform public health policies for the management and mitigation of influenza-like outbreaks in the community.

**Electronic supplementary material:**

The online version of this article (doi:10.1186/s12879-014-0695-9) contains supplementary material, which is available to authorized users.

## Background

It has been long known [1]-[3] that children play an important role in the community spread of infectious disease, in particular of influenza. The many contacts children have with one another at school increase their risk of being infected by several droplet-transmitted pathogens, and make schools an important source of transmission to households, from where the disease can spread further. For instance, during the 2009 H1N1 pandemic, a correlation was observed [4] between the opening dates of schools and the onset of widespread transmission of H1N1 in the US. Similarly, the timing of school terms, with the corresponding changes in contact patterns, has been shown to explain the evolution of the H1N1 epidemic in the UK [5].

School closure is thus regarded as a viable mitigation strategy for epidemics [6],[7], especially in the case of novel pandemics for which pharmaceutical interventions, such as vaccines, are not readily available and delaying disease spread is a priority. The impact of school closure on the spread of infectious disease has been studied using historical data [8]-[13], comparison of contact patterns during week days, weekends and holiday periods [5],[14],[15], and agent-based models at different scales [9],[16]-[19]. School closure, however, comes with a steep socio-economic cost, as parents need to take care of their children and might be forced to take time off work. This can even have a detrimental impact on the availability of public health staff. Such harmful side effects have led some to question the effective benefit of school closure [20],[21] and have prompted research on the design and evaluation of non-pharmaceutical low-cost mitigation strategies.

In this context, the availability of data on contacts between school children is a crucial asset on two accounts. First, even limited information on mixing patterns within and between classes or grades can suggest more refined strategies than whole-school closure. Second, high-resolution contact data allow the development of individual-based computational models of disease spread that can be used to test and compare different mitigation strategies. Because of this, over the last few years a great deal of effort has been devoted to gathering data on human contact patterns in various environments [22], using methods that include diaries and surveys [14],[23]-[31], and more recently wearable sensors that detect close-range proximity [32],[33] and face-to-face contacts [34]-[39].

In this study we use a high-resolution contact network measured by using wearable sensors in a primary school [38]. The data show that children spend more time in contact with children of the same class (on average three times more than with children of other classes) and of their own grade [38]. This is expected to be a rather general qualitative feature of schools, due both to age homophily [40] and schedule constraints, and suggests that transmission events might take place preferentially within the same class or grade^a^. We thus consider targeted and reactive mitigation strategies in which one class or one grade is temporarily closed whenever symptomatic individuals are detected. To evaluate the effectiveness of such micro-interventions we use our high-resolution contact network data [34],[38] to build an individual-based model of epidemic spread, and we compare, in simulation, the performance and impact on schooling of different targeted mitigation strategies with the closure of the whole school.

## Methods

### High-resolution contact network data

We use a high-resolution contact network measured by the SocioPatterns collaboration [34] using wearable proximity sensors in a primary school. The sensors detect the face-to-face proximity relations (“contacts”) of individuals with a 20-seconds temporal resolution [35]. The time-resolved contact network considered here, analyzed in Ref. [38], describes the contacts among 232 children and 10 teachers in a primary school in Lyon, France, and covers two days of school activity (Thursday, October 1^*st*^ and Friday, October 2^*nd*^ 2009). The school is composed by 5 grades, each of them comprising two classes, for a total of 10 classes. Contacts events are individually resolved, and their starting and ending times are known up to the 20-second resolution of the measurement system.

The French national bodies responsible for ethics and privacy, the Commission Nationale de l’Informatique et des Libertés (CNIL, http://www.cnil.fr) and the ‘Comité de Protection des personnes’ (http://www.cppsudest2.fr/), were notified of the study, which was approved by the relevant academic authorities (by the ‘directeur de l’enseignement catholique du diocèse de Lyon’, as the school in which the study took place is a private catholic school). In preparation for the study, parents and teachers were informed through an information leaflet, and were invited to a meeting in which the details and the aims of the study were illustrated. Verbal informed consent was then obtained from parents, teachers and from the director of the school. As no personal information of participants was collected, the relevant academic authorities considered that written consent was not needed. Special care was paid to the privacy and data protection aspects of the study: The communication between the sensors and the computer system used to collect data were fully encrypted. No personal information of participants was associated with the identifier of the corresponding sensor.

Daily-aggregated contact networks were published in the context of the original paper [38]. Here we make available to the public the full high-resolution dataset. We publish here as Additional file 1 the full time-resolved contact list, with node metadata on school role (students vs teachers) and class/grade affiliation of each individual.

### Extending the temporal span of the empirical data

Realistic parameters for the infectious and latent periods of influenza-like disease are of the order of days. Since the dataset we use only spans two school days, our numerical simulations will unfold over time scales longer than the duration covered by the contact dataset. To address this problem, several possibilities to extend in time the empirical contact data have been explored [41]. Here we consider a simple periodic repetition of the 2-day empirical data, modified to take into account specific features of the school environment under study. First, since our data only describes contacts during school hours, we assume that children are in contact with the general community for the rest of the day. Moreover, children in France do not go to school on Wednesday, Saturday and Sunday: on these days, therefore, children are also considered in contact with the general community. Overall the temporal contact patterns we use have the following weekly scheme:

i) Monday and Tuesday correspond to the first and second day of the empirical dataset: between 8.30am and 5:00pm contacts within the school are described by the empirical data. Outside of this interval, children are assumed to be isolated from one another and in contact with the community.
ii) Wednesday: children are in contact with the community for the entire day.
iii) Thursday and Friday: the first and second day of the empirical dataset are repeated as in i).
iv) Saturday and Sunday: children are in contact with the community for the entire weekend.

The above weekly sequence is repeated as many times as needed. Other extension procedures include partial reshuffling of the participants’ identities across days [41], to model the partial variability of each individual’s contacts from one day to the next. Here we limit our investigation to the simple scheme outlined above, because a repetition procedure is appropriate to model a school environment, where activities follow a rather repetitive daily and weekly rhythm, and each child is expected to interact every day with approximately the same set of individuals, namely the members of her/his class and her/his acquaintances in other classes.

### Epidemic model

To simulate the spread of an influenza-like disease we consider an individual-based stochastic SEIR model with asymptomatic individuals, with no births, nor deaths, nor introduction of individuals [42]. In such a model each individual at a given time can be in one of five possible states: susceptible (S), exposed (E), infectious and symptomatic (I), infectious and asymptomatic (A), and recovered (R). Whenever a susceptible individual is in contact with an infectious one, she/he can become exposed at rate *β* if the infectious individual is symptomatic, and *β*/2 if the infectious individual is asymptomatic [43]-[47]^b^. Exposed individuals, who cannot transmit the disease, become infectious after a latent period of average 1/*μ*. Exposed individuals becoming infectious have a probability *p*_*A*_ of being asymptomatic (A) and a probability 1−*p*_*A*_ of being symptomatic (I). Both symptomatic and asymptomatic infectious individuals recover at the end of the infectious period of average duration 1/*γ*, and acquire permanent immunity to the disease.

As mentioned above, our data describe human contacts only within the school premises. During the spread of an epidemic in the community, however, exposure to infectious individuals also occurs outside of school. Accordingly, we consider that individuals have a generic risk of being contaminated by infectious individuals outside of the school. For simplicity, here we assume that this risk is uniform and we introduce it into the model through a fixed rate of infection *β*_*com*_. That is, the probability that a susceptible individual, during a small time interval *dt*, becomes exposed due to random encounters outside of school is *β*_*com*_*dt*.

Finally, we assume that symptomatic individuals are detected at the end of each day. They are subsequently isolated until they recover and therefore cannot transmit the disease anymore. Asymptomatic individuals, on the other hand, cannot be detected and thus are not isolated. Each simulation starts with a completely susceptible population, except for a single, randomly chosen infectious individual, chosen as symptomatic with probability 1−*p*_*A*_ and asymptomatic with probability *p*_*A*_.

We consider the following parameter values for the SEIR model: *β*=3.5·10^−4^*s*^−1^ (1/*β*≈48*min*)^c^, *β*_*com*_=2.8·10^−9^*s*^−1^, 1/*μ*=2 days, 1/*γ*=4 days. As in many previous studies [43]-[47],[49] and in a way compatible with empirical results [49], the fraction of infected asymptomatic individuals is set to *p*_*A*_=1/3. These parameter values are in line with those commonly used in models of influenza-like illnesses [41],[44]-[46],[50],[51]. Moreover, for each infected individual, we extract at random the durations of her/his latency and infectious periods from Gaussian distributions of respective averages 1/*μ* and 1/*γ* and standard deviations equal to one tenth of their average. We perform simulations with a time step *dt* determined by the temporal resolution of the data set considered, namely 20 seconds.

We carry out sensitivity analyses with respect to our modelling choices and parameters. First, we consider a larger value of *β*_*com*_ while keeping fixed the values of the other parameters, to investigate the role of the generic risk of infection in the community. Second, we report in the Additional file 2 the results obtained with two different sets of parameters corresponding to faster spreading processes, namely: (i) *β*=6.9·10^−4^*s*^−1^ (1/*β*≈24*min*); *β*_*com*_=2.8·10^−9^*s*^−1^; 1/*μ*=1 day; 1/*γ*=2 days, *p*_*A*_=1/3, and (ii) *β*=1.4·10^−3^*s*^−1^ (1/*β*≈12*min*), *β*_*com*_=2.8·10^−9^*s*^−1^, 1/*μ*=0.5 day, 1/*γ*=1 day, *p*_*A*_=1/3. Moreover, we also show in the Additional file 2 results obtained by assuming a larger fraction of asymptomatic individuals, namely *p*_*A*_=1/2. Third, we consider in the Additional file 2 a different shape for the distributions of the latent and infectious periods, namely Weibull distributions of average values 1/*μ* and 1/*γ* and various shape parameters, corresponding to broader distributions.

### Mitigation measures

The baseline mitigation measure is given by the isolation of symptomatic children at the end of each day. We consider the three following additional strategies: whenever the number of symptomatic infectious individuals detected in any class reaches a fixed threshold,

(i) the class is closed for a fixed duration (“targeted class closure” strategy);
(ii) the class and the other class of the same grade are both closed for a fixed duration (“targeted grade closure” strategy);
(iii) the entire school is closed for a fixed duration (“whole school closure” strategy).

In all cases, the children affected by the closure are considered to be in contact with the community during the closure period – with the exception of detected infectious cases – and therefore they have a probability per unit time *β*_*com*_ of acquiring the disease. When the closure is over, the class (or grade) is re-opened and the corresponding children go back to school.

For benchmarking purposes, in the Additional file 2 we also consider strategies based on random class closures: whenever the number of symptomatic infectious individuals detected in any class reaches a fixed threshold,

(iv) one random class, different from the one in which symptomatic individuals are detected, is closed (“random class closure” strategy)
(v) the class and a randomly chosen one in a different grade are closed (“mixed class closure” strategy).

Note that during the course of an epidemic, in principle, several classes can be closed at the same time or successively, but once a class (or grade) is re-opened, we do not allow it to be closed again. Similarly, when using the whole-school closure strategy, we assume for simplicity that once the school is re-opened it cannot be closed again.

All of the closure strategies describe above depend on two parameters: the closure-triggering threshold, i.e., the number of symptomatic individuals required to trigger the intervention, and the duration of the closure. We will explore thresholds of 2 or 3 symptomatic individuals and closure durations ranging from 24 to 144 hours (from 1 to 6 days). Closure durations are specified in terms of absolute time: for instance, a 72 hours closure starting on a Thursday night spans the following Friday, Saturday and Sunday and ends on the next Monday morning.

### Simulation and analysis

For each set of model parameters for the SEIR model, and for each set of parameters of every mitigation strategy we simulate 5000 realizations of the epidemic process. We compare the performance of different strategies by measuring (i) the fraction of stochastic realizations that yield an attack rate (fraction of individuals affected by the disease) higher than 10*%*, and (ii) the average number of final cases in the population. We also quantify the burden of each strategy by computing the number of lost schools days, defined by adding up the number of school days missed by each class affected by the intervention. A closure of one class during a normal school day counts as 1 lost day, whereas the closure of the entire school counts as 10 lost days, as there are 10 classes in the school. We do not count Wednesdays and week-ends spanned by the closure interval.

## Results and discussion

Here we provide results corresponding to the parameter values *β*=3.5·10^−4^*s*^−1^ (1/*β*≈48*min*), *β*_*com*_=2.8·10^−9^*s*^−1^, 1/*μ*=2 days, 1/*γ*=4 days. The results for the other sets of parameters and other distributions of latent and infectious periods’ durations are qualitatively similar and are discussed in the Additional file 2.

In Table 1 we report the fraction of stochastic realizations that lead to an attack rate (AR) higher than 10*%*, for each mitigation strategy and each set of parameter values of the strategy (closure triggering threshold and closure duration). As a baseline, we also report the attack rate obtained when no closure is implemented, i.e., when the only mitigation measure is the isolation of symptomatic individuals at the end of each school day. Even when no closure strategy is implemented the majority of realisations (65.4*%*) do not lead to a large outbreak. The probability of a large outbreak is reduced by all of the closure strategies. We observe a larger reduction for smaller values of the closure-triggering threshold and for longer closure durations. On closing whole grades (2 classes) rather than individual classes we report a smaller percentage of realizations leading to large outbreaks.

**Table 1:** **Percentage of realizations leading to an attack rate higher than 10** ***%*** **, for different mitigation strategies and for various closure-triggering thresholds and closure durations**

Closure strategy	Targeted	Targeted	Whole
(threshold, duration)	class	grade	school
No closure	34.6	34.6	34.6
3, 24 h	30.5	29.7	26.0
3, 48 h	28.1	23.5	23.2
3, 72 h	23.4	18.4	14.8
3, 96 h	23.5	20.3	13.0
3, 120 h	20.1	17.3	7.5
3, 144 h	19.7	16.3	5.6
2, 24 h	28.6	27.0	22.9
2, 48 h	22.0	21.6	17.8
2, 72 h	17.4	16.2	14.4
2, 96 h	13.6	11.2	11.0
2, 120 h	10.2	7.2	3.2
2, 144 h	11.6	6.8	1.6

The baseline case given by the simple isolation of symptomatic children is indicated as “No closure”. Parameter values: *β* = 3.5 · 10^-4^ *s* ^-1^, *β*_com_ = 2.8 · 10^-9^ *s* ^-1^, 1/ *μ* = 2 days, 1/ *γ* = 4 days, *p*_A_ = 1/3.

In Table 2 we complement the above results by reporting, for each strategy and parameter choice, the final number of cases (averages and confidence intervals) for realizations leading to an attack rate larger than 10*%*. For small enough closure triggering thresholds and long enough closure durations, all closure strategies achieve a strong reduction of the final epidemic size. Strategies affecting more classes also have a stronger effect, but in those cases we observe large confidence intervals and large overlap of the epidemic sizes for different choices of the strategy parameters. In particular, for small closuretriggering thresholds the targeted class and targeted grade strategies yield reductions in the number of large outbreaks that are similar to those observed for the closure of the whole school.

**Table 2:** **Average final number of cases, computed for realizations leading to an attack rate higher than 10** ***%*** **, for different mitigation strategies and for various closure-triggering thresholds and closure durations**

Closure strategy	Targeted	Targeted	Whole
(threshold, duration)	class	grade	school
No closure	179 [149,203]	179 [149,203]	179 [149,203]
3, 24 h	162 [122,199]	166 [112,196]	170 [151,202]
3, 48 h	135 [48,197]	138 [40,188]	162 [43,199]
3, 72 h	101 [33,186]	103 [30,177]	146 [28,198]
3, 96 h	92 [29,184]	88 [26,169]	120 [27,195]
3, 120 h	75 [29,170]	62 [25,163]	67 [26,192]
3, 144 h	71 [26,168]	58 [24,161]	55 [25,180]
2, 24 h	165 [91,195]	170 [141,199]	173 [139,198]
2, 48 h	124 [32,179]	142 [35,191]	170 [62,199]
2, 72 h	96 [30,170]	113 [29,180]	149 [48,201]
2, 96 h	75 [27,152]	94 [26,184]	141 [31,196]
2, 120 h	69 [25,140]	73 [25,181]	133 [30,195]
2, 144 h	51 [27,111]	52 [26,138]	57 [25,192]

The baseline case given by the simple isolation of symptomatic children is indicated as “No closure”. In square brackets we provide the 5^th^ and 95^th^ percentiles. Parameter values: *β* = 3.5 · 10^-4^ *s* ^-1^, *β*_com_ = 2.8 · 10^-9^ *s* ^-1^, 1/ *μ* = 2 days, 1/ *γ* = 4 days, *p*_A_ = 1/3.

**Figure 1:**
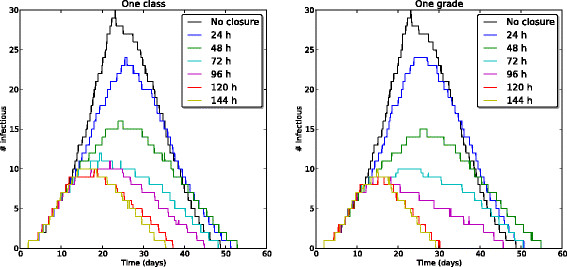
**Effect of closure duration.** Temporal evolution of the median number of infectious individuals. for several closure durations, at a fixed closure-triggering threshold of 3 symptomatic cases. Left: targeted class closure. Right: targeted grade closure. Only runs with an attack rate (AR) higher than 10*%* are taken into account. Parameter values: *β*=3.5·10^−4^ *s* ^−1^, *β*_*com*_=2.8·10^−9^ *s* ^−1^, 1/*μ*=2 days, 1/*γ*=4 days, *p*_*A*_=1/3.

**Figure 2:**
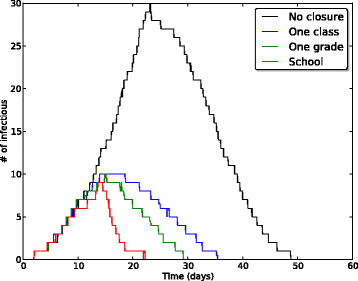
**Comparison of different strategies.** Temporal evolution of the median number of infectious individuals for the targeted class, targeted grade, and whole school closure strategies, at a fixed closure-triggering threshold of 3 infectious individuals and closure duration of 144 hours (6 days). The no-closure scenario is provided for reference. Only realizations with an attack rate (AR) higher than 10*%* are taken into account. Parameter values: *β*=3.5·10^−4^ *s* ^−1^, *β*_*com*_=2.8·10^−9^ *s* ^−1^, 1/*μ*=2 days, 1/*γ*=4 days, *p*_*A*_=1/3.

Figures 1 and 2 display the temporal evolution of the median number of infectious individuals for several mitigation strategies, when only realizations leading to an attack rate higher than 10*%* are considered. Figure 1 shows the effect of closure duration for the targeted class and targeted grade strategies at a fixed closure-triggering threshold of 3 symptomatic cases. Longer closures lead to shorter and smaller epidemic peaks. Closure durations of 5 or 6 days (120 and 144 hours, respectively) lead to very similar epidemic curves. Figure 2, on the other hand, compares the epidemic curves for the targeted class, targeted grade and whole school strategies at fixed closure-triggering threshold and closure duration parameters. The targeted class closure strategy already yields a large reduction of the epidemic peak, and this reduction is only slightly improved by the targeted grade closure and whole school closure strategies (for the same closure durations).

Finally, in Table 3 we report the impact on the schooling system of each closure strategy, quantified by the average number of lost school days aggregated over all affected classes. In all cases, a high percentage of realizations lead to zero impact, corresponding to (i) situations in which the outbreak stays confined and the closure-triggering threshold is never reached in any class, or (ii) to cases in which the closure happens on days during which the school is scheduled to be closed (Wednesdays or week-ends). Whenever schooldays are effectively lost, we observe a much greater impact for the whole school closure than for the alternative strategies of closing one class or one grade only.

**Table 3:** **Number of lost school days for the various closure strategies**

Closure strategy (Threshold, duration)	Targeted class	Targeted grade	Whole school
No closure	0	0	0
3, 24 h	2.14 (3.27) [0-9]	2.34 (3.58) [0-10]	2.50 (4.33) [0-10]
3, 48 h	3.04 (4.83) [0-13]	3.00 (5.12) [0-14]	4.42 (7.26) [0-20]
3, 72 h	3.01 (5.17) [0-15]	3.21 (5.44) [0-16]	5.38 (8.13) [0-20]
3, 96 h	4.49 (7.62) [0-22]	4.93 (8.21) [0-24]	8.10 (11.5) [0-30]
3, 120 h	4.50 (8.41) [0-26]	5.20 (9.06) [0-28]	9.38 (13.4) [0-40]
3, 144 h	4.67 (8.72) [0-27]	5.33 (9.41) [0-28]	9.60 (13.7) [0-40]
2, 24 h	2.18 (3.36) [0-9]	2.31 (3.46) [0-10]	3.38 (4.73) [0-10]
2, 48 h	2.42 (4.37) [0-13]	3.05 (4.77) [0-14]	4.60 (7.16) [0-20]
2, 72 h	2.57 (4.77) [0-15]	3.44 (5.45) [0-16]	7.12 (8.54) [0-20]
2, 96 h	3.14 (6.11) [0-20]	3.92 (6.80) [0-22]	8.64 (11.2) [0-30]
2, 120 h	3.55 (6.37) [0-18]	4.38 (7.68) [0-22]	9.32 (12.4) [0-30]
2, 144 h	4.32 (7.38) [0-22]	4.63 (7.61) [0-22]	11.54 (13.7) [0-40]

The baseline case in which no class is closed (“No closure”) has a zero cost in terms of lost school days. Standard deviations are given in parentheses and 5^th^ and 95^th^ percentiles in square brackets. Parameter values: *β* = 3.5 · 10^-4^ *s* ^-1^, *β*_com_ = 2.8 · 10^-9^ *s* ^-1^, 1/ *μ* = 2 day, 1/ *γ* = 4 days, *p*_A_ = 1/3.

### Effect of the risk of infection in the community

As mentioned in the Methods section, to assess the role of the risk of infection due to contacts in the community (as opposed to those at school), we consider a set of parameter values where *β*_*com*_ is increased five-fold with respect to the previous results. That is, we use the values *β*=3.5·10^−4^*s*^−1^, *β*_*com*_=1.4·10^−8^*s*^−1^, 1/*μ*=2 days, 1/*γ*=4 days, *p*_*A*_=1/3. Tables 4 and 5 report the results we obtain with this higher value of *β*_*com*_ for the targeted closure strategies. The probability of a large outbreak is much higher than in the previous case (as shown by comparing Table 4 with Table 1). This probability is reduced by the targeted closure strategies, but remains comparatively large. As observed for the smaller *β*_*com*_, the decrease in the probability of a large outbreak is larger for longer closure durations, for smaller closure-triggering thresholds, and for closures involving more classes.

**Table 4:** **Percentage of realizations leading to an attack rate higher than 10** ***%*** **, for the different mitigation strategies with several closure-triggering thresholds and closure durations**

Closure strategy	Targeted	Targeted	Whole
(threshold, duration)	class	grade	school
No closure	65.9	65.9	65.9
3, 24 h	65.0	62.4	64.1
3, 48 h	58.7	59.0	58.9
3, 72 h	58.0	57.4	53.7
3, 96 h	58.1	56.3	44.0
3, 120 h	56.4	51.2	38.9
3, 144 h	55.3	51.0	38.7
2, 24 h	65.7	59.0	60.7
2, 48 h	57.3	56.1	53.2
2, 72 h	49.7	49.5	45.3
2, 96 h	46.7	46.2	43.3
2, 120 h	44.0	37.7	35.9
2, 144 h	41.5	36.8	31.7

The baseline case given by the simple isolation of symptomatic children is indicated as “No closure”. Parameter values: *β* = 3.5 · 10^-4^ *s* ^-1^, *β*_com_ = 1.4 · 10^-8^ *s* ^-1^, 1/ *μ* = 2 days, 1/ *γ* = 4 days, *p*_A_ = 1/3.

**Table 5:** **Average final number of cases for realizations leading to an attack rate higher than 10** ***%*** **, for different mitigation strategies**

Closure strategy	Targeted	Targeted	Whole
(threshold, duration)	class	grade	school
No closure	187 [161,192]	187 [161,192]	187 [161,192]
3, 24 h	173 [142,196]	176 [155,200]	181 [153,200]
3, 48 h	155 [114,185]	157 [69,191]	178 [143,203]
3, 72 h	131 [52,170]	137 [40,188]	169 [37,200]
3, 96 h	118 [100,182]	120 [28,182]	161 [30,200]
3, 120 h	103 [35,164]	100 [29,174]	145 [28,196]
3, 144 h	102 [39,161]	89 [28,173]	126 [26,196]
2, 24 h	176 [145,201]	177 [149,202]	183 [160,203]
2, 48 h	151 [89,186]	158 [89,196]	180 [155,201]
2, 72 h	118 [37,177]	136 [32,189]	179 [149,201]
2, 96 h	111 [30,180]	120 [31,191]	176 [141,202]
2, 120 h	102 [27,168]	106 [27,188]	176 [155,198]
2, 144 h	93 [28,167]	94 [27,185]	174 [88,205]

The baseline case given by the simple isolation of symptomatic children is indicated as “No closure”. In square brackets we provide the 5^th^ and 95^th^ percentiles. Parameter values: *β* = 3.5 · 10^-4^ *s* ^-1^, *β*_com_ = 1.4 · 10^-8^ *s* ^-1^, 1/ *μ* = 2 days, 1/ *γ* = 4 days, *p*_A_ = 1/3.

**Figure 3:**
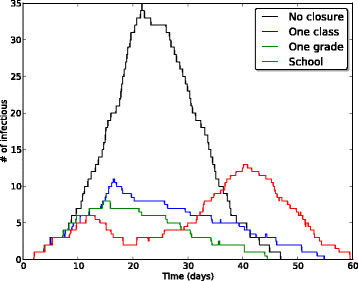
**Case of a higher risk of infection in the community.** Temporal evolution of the median number of infectious individuals, for the targeted class and targeted grade closure strategies with a closure-triggering threshold of 3 infectious individuals and a closure duration of 144 hours (6 days), compared with the scenario without closure and the whole school closure strategy with a closure duration of 144 hours. Here *β*=3.5·10^−4^ *s* ^−1^, *β*_*com*_=1.4·10^−8^ *s* ^−1^, 1/*μ*=2 days, 1/*γ*=4 days, *p*_*A*_=1/3. Only realizations with attack rate (AR) larger than 10*%* are taken into account.

In the case of epidemics reaching more than 10*%* of the population, however, Table 5 shows that the targeted class and targeted grade closure strategies lead to smaller attack rates than the whole school closure strategy. Figure 3 gives more insight into this point by showing the epidemic curve for realizations with a final attack rate larger than 10%, for the targeted and school closure strategies with a closure duration of 144 hours and a closure-triggering threshold of 3 infectious individuals. The effect of the targeted class and grade closure strategies is similar to the case of a smaller *β*_*com*_: these strategies lead to a smaller and shorter epidemic peak with respect to the baseline strategy. The epidemic curve for the whole school closure strategy on the other hand is changed and has now two successive peaks; even if the first one is smaller than for the targeted strategies, the presence of the second peak, due to the end of the school closure, leads overall to a larger final attack rate. Such poor performance of the whole school closure might thus occur because the intervention is being applied and relaxed too early and because of its *a priori* limited duration.

## Conclusions

Since the contacts of children at school play an important role for the propagation of many infectious diseases in the community, it is crucial to devise efficient and cost-effective mitigation strategies as an alternative to the closure of whole schools, whose socio-economic costs are often considered excessive. Inspired by the empirical evidence collected in European primary and high schools [28],[38],[52] that children did not mix homogeneously but rather spent much more time in contact with their classmates and with other children of the same age, we have designed targeted closure strategies at the class or grade level that are reactively triggered when symptomatic cases are detected. We have simulated the dynamics of epidemic spread among school children by using an SEIR model on top of a high-resolution time-resolved contact network measured in a primary school. The model included asymptomatic individuals and a generic risk of infection due to random contacts with the community when children are not at school. Using this model we have studied the targeted strategies for class and grade closure both in terms of their ability to mitigate the epidemic and in terms of their impact on the schooling system (and therefore indirectly on the whole community), measured by the number of cancelled days of class.

All targeted strategies lead to an important reduction in the probability of an outbreak reaching a large fraction of the population. In the case of large outbreaks, targeted strategies significantly reduce the median number of individuals affected by the epidemic. The reduction is stronger if the strategies are triggered by a smaller number of symptomatic cases, and if longer closures durations are used. While the closure of one class yields a smaller mitigation effect than the closure of the whole school, the closure of the corresponding grade (two classes) leads to a reduction of large outbreak probability and a reduction of epidemic size that are similar to those obtained by closing the entire school. The less efficient results of the benchmark strategies (iv) and (v) involving closure of randomly chosen classes (given in the Additional file 2) also show that the effect of the class-targeting strategies is due to the targeting of contacts of cases, and not just to the size of the closure (i.e., the number of children involved by the closure). Targeted strategies, moreover, come at a much smaller cost in terms of lost class days. Such decrease in the cost of the intervention might also imply a smaller global impact of the spread on the whole community. In the case of large outbreaks and large risk of infection in the community, whole-school closure might even lead to a smaller mitigation effect than targeted grade closure, as more susceptible children would spend more time in the community, acquiring the infection and subsequently bringing it back into the school upon re-opening.

A few important points need to be stressed. First, the reactive character of all strategies we studied, which are triggered by the detection of symptomatic individuals, limits the impact on the schooling system with respect to a closure of schools scheduled in a top-down fashion by public health authorities: the latter would be enforced even for schools that are free of infectious individuals. Second, targeted grade closure has in all cases a much lighter burden, in terms of lost class days, than whole-school closure. Given also its good performance in the mitigation of outbreaks, it thus represents an interesting alternative strategy. Finally, we recall that grade closure corresponds to closing the class in which symptomatic children are detected and the class which has the most contacts with it. To assess this relation between classes, we do not need the very detailed knowledge of the contact patterns we used in this study: rather, readily available information such as class schedules and classroom locations [53] may be sufficient to retrieve this information. This has important public health consequences, as it implies that the targeted mitigation strategies studied here might actually be carried out in the general case, without high-resolution contact network data.

Some limitations of this study are worth mentioning. While the strategies we discussed could be designed and implemented with limited information, they were only tested using one specific dataset corresponding to one particular school. Different schools, either concerning different ages (e.g., high schools [36],[52]) or in different countries, might lead to either more or less structured contacts between children or students of different classes. The high-resolution contact network data we used only spans two days of school activity, and had to be extended longitudinally by using a repetition procedure. This technique is commonly used to simulate epidemic spread on temporal data, but it does introduce strong temporal correlations in the extended dataset and it may fail to correctly model the day-to-day heterogeneity of contact patterns [41]. While variations in the repetition of contacts from one day to the next are known to modify the attack rate of an epidemic [50], we expect that the relative efficiency of the strategies we considered should be robust with respect to other temporal extensions strategies [41]^d^. Moreover, this limitation should be less of an issue in school settings, where mixing patterns are shaped by a regular activity schedule and have a strong periodic character.

Another limitation of this study is the simplistic coupling with the community that we used: our high-resolution contact network does not include contacts happening outside of school, so we introduced in our model a free parameter that describes a generic risk of transmission from the community. Even though our results are robust with respect to important variations in this parameter, it would be desirable to inform the model with empirical data on the contacts that children have with members of the community, or with one another outside of school. Moreover, while we have established the effectiveness of the class closure measures in reducing the spread within the school population, the effect of these measures on the spread in the rest of the population is not modelled nor quantified. In fact, effective interventions at the school level might even have a further beneficial effect by leading to a decrease of the spread in the whole community, given the role of children in such spread.

The limitations described above point to several directions for further research. It would be interesting to validate the targeted class and grade closure strategies using high-resolution data describing the contacts of children in other schools and over longer timescales, if such datasets become available in the future. In particular it would be interesting to consider larger schools, for which the closure of more than two classes may represent an efficient intervention. An expansion of the model in order to refine the coupling with the community would also represent an important step, on the one hand to check that the simplistic coupling used here does not impact our results, and on the other hand to quantify the possible feedback effect of the mitigation of the spread within the school on the spread in the community. To this aim, high-resolution measurement of contact patterns within a school could for instance be coupled with surveys administered to the same children, to estimate their contact rates off-school and to model their contacts with other individuals of different age classes in the community. Such data could be used to refine the model used here, but also to design agent-based models at a larger scale (e.g., urban or geographic), spanning several schools [19]. This would allow to generalize the strategies introduced in this paper to the case of multiple schools, and to evaluate their relative efficiency, in particular comparing targeted strategies with the general closure of all schools in the relevant geographical region. Finally, we have discussed how targeted interventions can be guided by readily-available information on the school activities and organisational structure. New techniques to tease apart meso-scale activity patterns in high-resolution contact data [54] could be used to design and guide targeted intervention aimed not just at closing classes but, for example, at suspending or modifying specific activities in the school that involve the shared use of spaces (e.g., sports activities, time in the playground, lunch at the cafeteria, etc.).

## Endnotes

^a^The quantitative extent of this effect might depend on school specificities and in particular might differ between primary schools and high schools, or between countries [28],[36].

^b^if a susceptible is in contact with *n* infectious individuals, the forces of infection add as each possible transmission event is evaluated independently.

^c^We consider values of *β* close to the ones used in [41],[48], which lead to a reproductive number *R*_0_ close to 1.5.

^d^An indication that this is indeed the case is given by the fact that additional simulations, in which we consider only one of the two days of data and repeat it yield very similar (qualitative and quantitative) results.

## Additional files

## Electronic supplementary material

Additional file 1: Temporal network data. This file contains the temporal network of contacts between the children and teachers used in the present study. The file contains a tab-separated list representing the active contacts during 20-second intervals of the data collection. Each line has the form “t i j Ci Cj”, where i and j are the anonymous IDs of the persons in contact, Ci and Cj are their classes, and the interval during which this contact was active is [ t - 20s, t ]. If multiple contacts are active in a given interval, you will see multiple lines starting with the same value of t. Time is measured in seconds. (TXT 3 MB)

Additional file 2: Supplementary Text. Additional text describing (i) the results obtained with different set of parameters for the epidemic model and (ii) the comparison between targeted and random class closures. (PDF 467 KB)

Below are the links to the authors’ original submitted files for images.

Authors’ original file for figure 1

Authors’ original file for figure 2

Authors’ original file for figure 3
