# Supplementary material for: Mitigation of infectious disease at school: targeted class closure vs school closure
Source: BMC Infect Dis. 2014 Dec 31;14:695. doi: 10.1186/s12879-014-0695-9 (PMC4297433; doi:10.1186/s12879-014-0695-9)
Supplement: Supplementary file 2 — Additional file 2: Supplementary Text. Additional text describing (i) the results obtained with different set of parameters for the epidemic model and (ii) the comparison between targeted and random class closures. (PDF 467 KB) [file 12879_2014_695_MOESM2_ESM.pdf]

Mitigation of infectious disease at school: targeted class closures vs school closures Supplementary Text

V. Gemmetto,[1] A. Barrat,[2,1] and C. Cattuto[1]

[1]*Data Science Laboratory, Institute for Scientific Interchange (ISI) Foundation, Torino, Italy*
[2]*Aix Marseille Université, Université de Toulon,*
CNRS, CPT, UMR 7332, 13288 Marseille, France

RESULTS OF THE SPREADING SIMULATIONS FOR MODIFIED SEIR MODEL PARAMETERS

As mentioned in the main text, we report here the results of numerical simulations performed with different sets of parameters for the SEIR epidemic model, in order to assess the robustness of the obtained results. We use here

(i) $\beta = 6.9 \cdot 10^{-4} s^{-1}$, $\beta_{com} = 2.8 \cdot 10^{-9} s^{-1}$, $1/\mu = 1$ day, $1/\gamma = 2$ days, $p_A = 1/3$;

(ii) $\beta = 1.4 \cdot 10^{-3} s^{-1}$, $\beta_{com} = 2.8 \cdot 10^{-9} s^{-1}$, $1/\mu = 0.5$ day; $1/\gamma = 1$ day, $p_A = 1/3$;

(iii) $\beta = 3.5 \cdot 10^{-4} s^{-1}$, $\beta_{com} = 2.8 \cdot 10^{-9} s^{-1}$, $1/\mu = 2$ days; $1/\gamma = 4$ days, $p_A = 1/2$.

Cases (i) and (ii) correspond to faster processes with respect to the parameters used in the main text, at fixed β_{com}, fixed ratio β/μ and fixed fraction of asymptomatic individuals: As the average latent and infectious periods are shorter than for the simulations presented in the main text, the epidemic will unfold here on shorter timescales. Case (iii) on the other hand corresponds to a larger fraction of asymptomatic individuals.

We implement the same mitigation strategies as in the main text, and, in order to compare their relative efficiencies, we focus on the fraction of stochastic realizations that yield a global attack rate higher than 10%, on the final average number of cases and on the temporal evolution of the number of infectious individuals. We compare the results obtained by implementing the various mitigation measures with the baseline case, represented by the situation in which the only mitigation strategy consists in the isolation of the symptomatic individuals once they are detected.

Results

In Tables I, II and III we show the fraction of stochastic realizations leading to an attack rate (AR) higher than 10%, for various mitigation measures, compared with the same result obtained when no closure was implemented. Compared with the results presented in the main text, faster spread lead to a decrease in the probability of large outbreaks even in the baseline case, while larger fractions of asymptomatic individuals make large outbreaks more probable.

Overall however, the same phenomenology is observed for all parameter values: the reduction of the probability of a severe outbreak occurs for all strategies, and increases for smaller triggering thresholds and longer closure durations. The closure of the whole school always leads to the most important effect, but the grade closure has as well a strong impact on the probability of occurrence of large outbreaks.

Tables IV, V and VI give the final number of cases for the parameter sets (i), (ii) and (iii), computed for the realisations leading to an attack rate larger than 10% for the various mitigation strategies. Faster spread lead to smaller number of cases, while increasing p_A has the opposite effect. Overall, all strategies lead to a reduction in the final number of cases. Interestingly, and similarly to the case shown in the main text, this reduction is very similar for the various closures (class, grade or whole school) if the closure triggering threshold is small enough: in cases of large outbreaks, closing only one class can be as effective as closing the whole school. As in the main text, we also note that large confidence intervals are observed, limiting the predictability of the final number of cases. Finally, as the duration of the closures is increased, the impact on the spread saturates at a value close to the sum of the latent and infectious periods, as in the main text.

Closure strategy (Threshold, duration)	Targeted class	Targeted grade	Whole school
No closure	26.4	26.4	26.4
3, 24 h	22.8	21.8	21.7
3, 48 h	21.8	17.0	13.3
3, 72 h	18.6	15.8	6.3
3, 96 h	16.6	14.1	5.4
2, 24 h	22.2	21.5	19.7
2, 48 h	16.8	17.0	12.6
2, 72 h	11.9	11.2	3.4
2, 96 h	10.2	8.7	3.5

TABLE I. Percentage of realizations leading to an attack rate higher than 10%, for the various mitigation strategies with various thresholds and closure durations. The baseline case given by the simple isolation of symptomatic children is indicated as "No closure". $\beta = 6.9 \cdot 10^{-4} s^{-1}$, $\beta_{com} = 2.8 \cdot 10^{-9} s^{-1}$, $1/\mu = 1$ day, $1/\gamma = 2$ days, $p_A = 1/3$.

Closure strategy (Threshold, duration)	Targeted class	Targeted grade	Whole school
No closure	13.4	13.4	13.4
3, 24 h	10.0	8.6	6.9
3, 48 h	7.3	6.7	3.2
3, 72 h	8.8	5.9	4.3
2, 24 h	7.4	6.0	4.4
2, 48 h	5.6	3.1	2.3
2, 72 h	5.3	3.2	1.9

TABLE II. Percentage of realizations leading to an attack rate higher than 10%, for the various mitigation strategies with various thresholds and closure durations. The baseline case given by the simple isolation of symptomatic children is indicated as "No closure". Here $\beta = 1.4 \cdot 10^{-3} s^{-1}$, $\beta_{com} = 2.8 \cdot 10^{-9} s^{-1}$, $1/\mu = 0.5$ day, $1/\gamma = 1$ day, $p_A = 1/3$.

Closure strategy (Threshold, duration)	Targeted class	Targeted grade	Whole school
3, 24 h	48.2	47.9	45.4
3, 48 h	45.2	43.6	41.3
3, 72 h	42.4	41.4	33.2
3, 96 h	41.4	40.2	30.1
3, 120 h	41.0	38.2	23.2
3, 144 h	40.6	37.6	19.8
2, 24 h	47.7	42.6	41.2
2, 48 h	41.8	39.0	37.2
2, 72 h	38.2	31.4	25.1
2, 96 h	34.8	29.7	22.8
2, 120 h	31.2	24.8	13.4
2, 144 h	28.8	21.2	9.3

TABLE III. Percentage of realizations leading to an attack rate higher than 10%, for the various mitigation strategies with various thresholds and closure durations. The baseline case given by the simple isolation of symptomatic children is indicated as "No closure". Here $\beta = 3.5 \cdot 10^{-4} s^{-1}$, $\beta_{com} = 2.8 \cdot 10^{-9} s^{-1}$, $1/\mu = 2$ days, $1/\gamma = 4$ days, $p_A = 1/2$.

Closure strategy (Threshold, duration)	Targeted class	Targeted grade	Whole school
No closure	154 [106,209]	154 [106,209]	154 [106,209]
3, 24 h	133 [91,194]	131 [90,192]	141 [101,206]
3, 48 h	100 [68,175]	99 [68,174]	111 [72,179]
3, 72 h	71 [39,135]	63 [37,133]	57 [35,129]
3, 96 h	66 [38,124]	58 [37,122]	38 [21,89]
2, 24 h	130 [88,192]	126 [83,189]	141 [103,201]
2, 48 h	94 [67,169]	91 [66,169]	124 [99,197]
2, 72 h	54 [36,120]	52 [36,119]	53 [34,126]
2, 96 h	51 [37,109]	45 [35,104]	43 [33,107]

TABLE IV. Average final number of cases, computed for the realisations leading to an attack rate larger than 10% for the various mitigation strategies; the brackets provide the 5th and 95th percentiles. The baseline case given by the simple isolation of symptomatic children is indicated as "No closure". $\beta = 6.9 \cdot 10^{-4} s^{-1}$, $\beta_{com} = 2.8 \cdot 10^{-9} s^{-1}$, $1/\mu = 1$ day, $1/\gamma = 2$ days, $p_A = 1/3$.

Closure strategy (Threshold, duration)	Targeted class	Targeted grade	Whole school
No closure	61 [26,128]	61 [26,128]	61 [26,128]
3, 24 h	53 [26,98]	51 [27,100]	55 [26,101]
3, 48 h	45 [26,88]	43 [26,68]	39 [25,70]
3, 72 h	45 [26,86]	41 [26,67]	34 [25,48]
2, 24 h	48 [25,85]	47 [26,81]	51 [26,104]
2, 48 h	39 [25,64]	38 [26,63]	41 [26,81]
2, 72 h	38 [26,64]	36 [25,51]	35 [25,48]

TABLE V. Average final number of cases, computed for the realisations leading to an attack rate larger than 10%, for the various mitigation strategies; the brackets provide the 5th and 95th percentiles. The baseline case given by the simple isolation of symptomatic children is indicated as "No closure". Here $\beta = 1.4 \cdot 10^{-3} s^{-1}$, $\beta_{com} = 2.8 \cdot 10^{-9} s^{-1}$, $1/\mu = 0.5$ day, $1/\gamma = 1$ day, $p_A = 1/3$.

Closure strategy (Threshold, duration)	Targeted class	Targeted grade	Whole school
No closure	195 [176,210]	195 [176,210]	195 [176,210]
3, 24 h	185 [164,205]	188 [167,207]	192 [173,209]
3, 48 h	158 [71,192]	162 [92,198]	184 [64,209]
3, 72 h	139 [44,183]	138 [38,187]	165 [28,203]
3, 96 h	136 [51,176]	122 [31,184]	141 [28,204]
3, 120 h	121 [37,168]	100 [31,171]	104 [29,200]
3, 144 h	115 [42,168]	89 [29,165]	70 [26,195]
2, 24 h	187 [168,205]	190 [166,207]	193 [178,210]
2, 48 h	156 [70,192]	169 [95,200]	188 [171,207]
2, 72 h	139 [48,186]	151 [34,195]	181 [146,207]
2, 96 h	120 [33,180]	130 [30,191]	175 [164,204]
2, 120 h	87 [26,170]	92 [26,183]	143 [30,206]
2, 144 h	79 [29,162]	74 [27,180]	87 [25,204]

TABLE VI. Average final number of cases, computed for the realisations leading to an attack rate larger than 10%, for the various mitigation strategies; the brackets provide the 5th and 95th percentiles. The baseline case given by the simple isolation of symptomatic children is indicated as "No closure". Here $\beta = 3.5 \cdot 10^{-4} s^{-1}$, $\beta_{com} = 2.8 \cdot 10^{-9} s^{-1}$, $1/\mu = 2$ days, $1/\gamma = 4$ days, $p_A = 1/2$.

Figures 1 and 2 complement the results by showing the temporal evolution of the median number of infectious individuals, for realisations leading to an attack rate larger than 10%.

Figure 1 shows the effect of various closure durations, for a closure triggering threshold of three symptomatic individuals and for the targeted class and grade closure strategies. For larger β, μ and γ, the epidemic curves unfold on shorter time-scales than in the case shown in the main text, as expected. As a result, the various strategies do not change much the epidemic peak timing, and have a smaller influence on the global duration of the spread, especially for the fastest spread. Moreover, in both cases, very similar epidemic curves are obtained when the closure duration becomes larger than the sum of $1/\mu$ and $1/\gamma$, while shorter durations yield a smaller effect. The optimal closure duration is thus close to the sum of the latent and infectious periods.

Figure 2 finally compares the epidemic curves for the targeted class closure, targeted grade closure, and whole school closure strategies, at fixed closure duration and closure-triggering threshold.

Overall, these results represent a very similar phenomenology with respect to the parameters used in the simulations shown in the main text, indicating the robustness of our results with respect to changes in the disease parameters and the relevance of targeted class and grade strategies as alternative measures to the closure of whole schools.

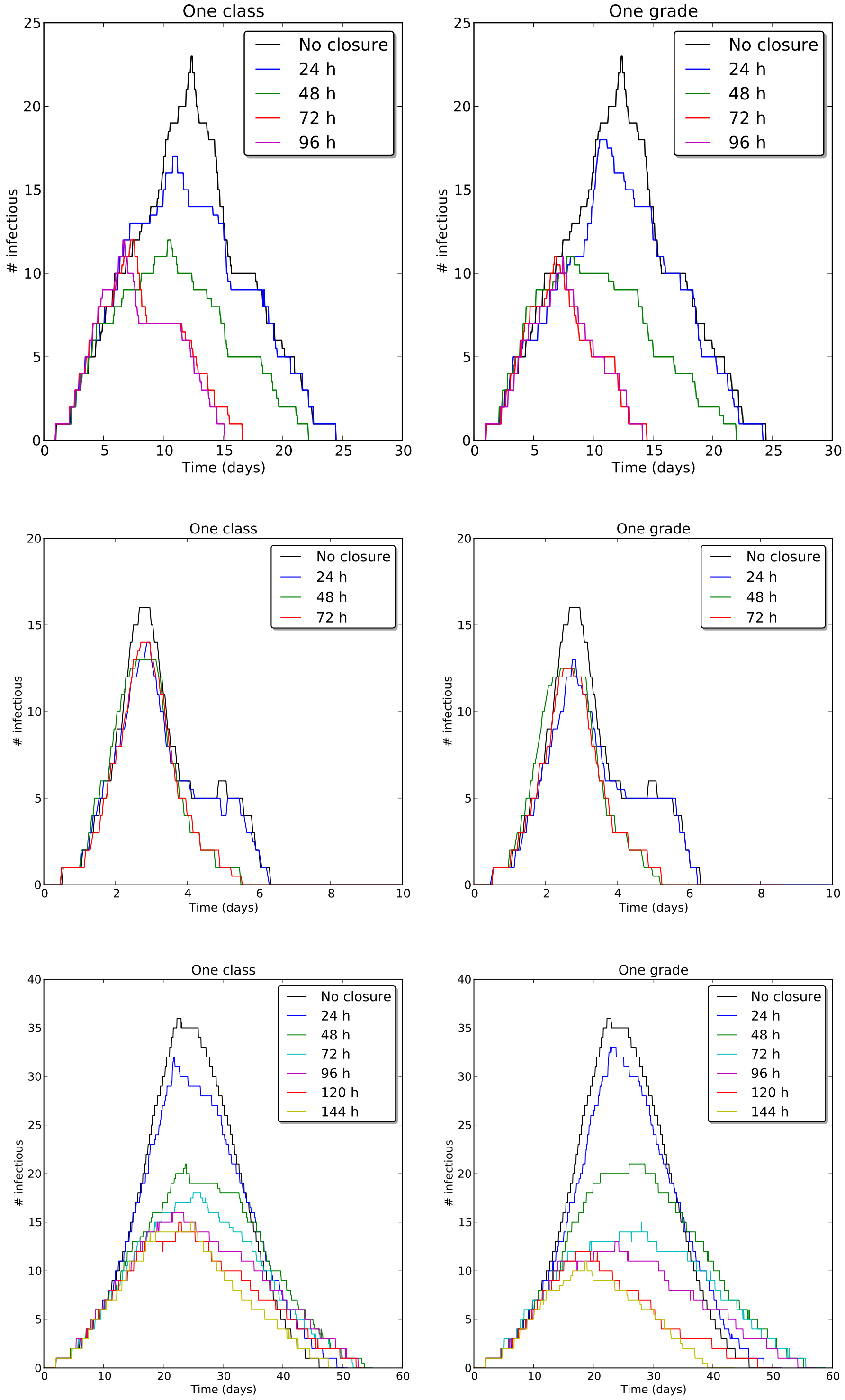

FIG. 1. Temporal evolution of the median number of infectious individuals, for various closure durations, in the case of a closure-triggering threshold equal to 3. Left: targeted class closure; right: targeted grade closure. Top plots: $\beta = 6.9 \cdot 10^{-4} s^{-1}$, $\beta_{com} = 2.8 \cdot 10^{-9} s^{-1}$, $1/\mu = 1$ day, $1/\gamma = 2$ days, $p_A = 1/3$. Middle plots: $\beta = 1.4 \cdot 10^{-3} s^{-1}$, $\beta_{com} = 2.8 \cdot 10^{-9} s^{-1}$, $1/\mu = 0.5$ day, $1/\gamma = 1$ day, $p_A = 1/3$. Bottom plots: $\beta = 3.5 \cdot 10^{-4} s^{-1}$, $\beta_{com} = 2.8 \cdot 10^{-9} s^{-1}$, $1/\mu = 2$ days, $1/\gamma = 4$ days, $p_A = 1/2$. Only runs with attack rate (AR) larger than 10% are taken into account.

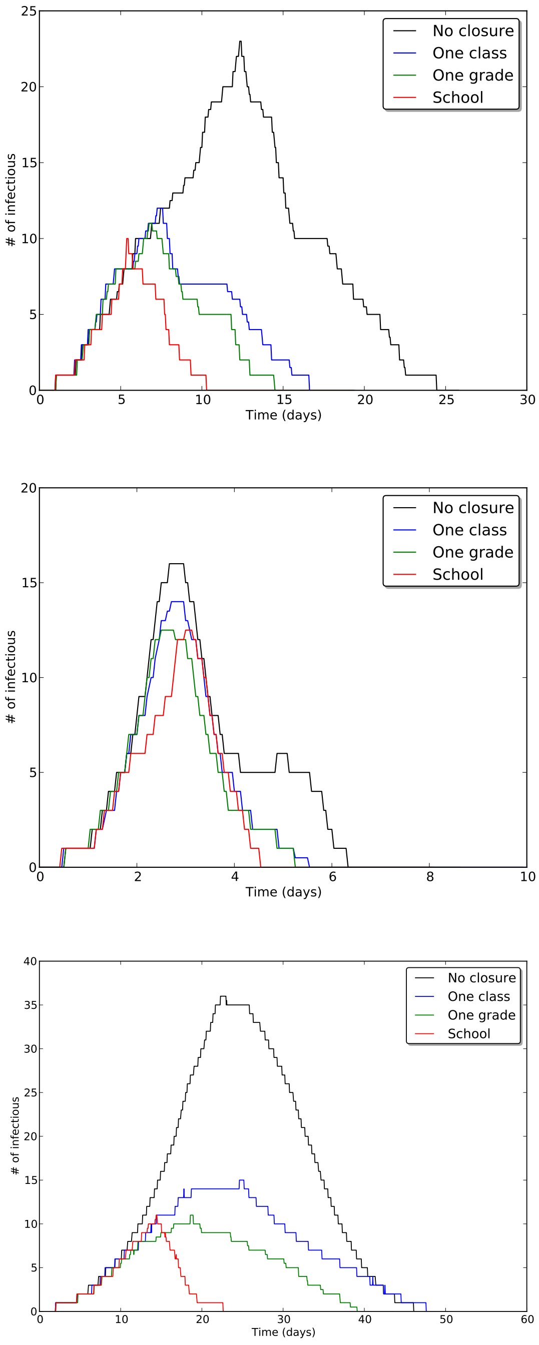

FIG. 2. Temporal evolution of the median number of infectious individuals, for the targeted class and targeted grade closure strategies with a closure-triggering threshold of 3 infectious individuals and a closure duration of H hours, compared with the scenario without closure and the whole school closure strategy with the same closure duration. Top: $\beta = 6.9 \cdot 10^{-4} s^{-1}$, $\beta_{com} = 2.8 \cdot 10^{-9} s^{-1}$, $1/\mu = 1$ day, $1/\gamma = 2$ days, $p_A = 1/3$, $H = 72$. Middle: $\beta = 1.4 \cdot 10^{-3} s^{-1}$, $\beta_{com} = 2.8 \cdot 10^{-9} s^{-1}$, $1/\mu = 0.5$ day, $1/\gamma = 1$ day, $p_A = 1/3$, $H = 72$. Bottom: $\beta = 3.5 \cdot 10^{-4} s^{-1}$, $\beta_{com} = 2.8 \cdot 10^{-9} s^{-1}$, $1/\mu = 2$ days, $1/\gamma = 4$ days, $p_A = 1/2$, $H = 144$. Only realizations with attack rate (AR) larger than 10% are taken into account.

WEIBULL-DISTRIBUTED DURATIONS OF THE LATENT AND INFECTIOUS PERIODS

In the main text and in the results shown above, the durations of the latent and infectious periods are taken at random from Gaussian distributions of averages $1/\mu$ and $1/\gamma$. These distributions have a standard deviation equal to one tenth of their average, meaning that the effective durations are rather close to the average values.

In order to check that our results are not sensitive to the specific distribution of latent and infectious periods distributions, we present here results obtained with other distributions, namely Weibull distributions of average $1/\mu$ for the latent period and $1/\gamma$ for the infectious period, respectively. We consider distributions of parameter shape $k = 3$ and $k = 4$, corresponding respectively to standard deviations equal approximately to 0.28 (for $k = 4$) and 0.36 (for $k = 3$) times the average (e.g., for $k = 3$ and an average of 2 days, the standard deviation is of 17.4 hours). These distributions are shown alongside the Gaussian distribution used in the main text in Figure 3

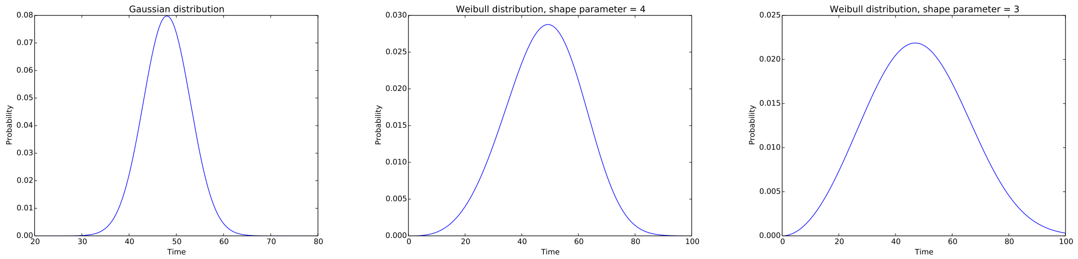

FIG. 3. Distributions of the latency period used in the simulations with $1/\mu = 2$ days. Left: Gaussian distribution. Centre: Weibull distribution with parameter shape $k = 4$. Right: Weibull distribution with parameter shape $k = 3$.

Tables VII-X and Figures 4-5 show that the percentages of runs leading to large outbreaks and the outbreak sizes depend slightly on the choice of the distribution of latent and infectious periods, but that the results remain qualitatively similar to the ones described in the main text.

Closure strategy (threshold, duration)	Targeted class	Targeted grade	Whole school
No closure	30.9	30.9	30.9
3, 24 h	29.1	27.7	27.4
3, 48 h	24.9	24.0	20.4
3, 72 h	22.7	22.3	18.5
3, 96 h	22.4	19.7	14.0
3, 120 h	19.9	17.4	10.4
3, 144 h	20.0	17.1	9.2
2, 24 h	28.1	27.4	25.2
2, 48 h	22.0	21.8	18.7
2, 72 h	18.3	16.9	14.0
2, 96 h	16.5	14.6	10.2
2, 120 h	15.0	10.1	8.3
2, 144 h	11.2	8.3	4.6

TABLE VII. Percentage of realizations leading to an attack rate higher than 10%, for the various mitigation strategies with various thresholds and closure durations. Parameter values (durations distributed according to Weibull distributions of shape parameter 3): $\beta = 3.5 \cdot 10^{-4}\, s^{-1}$, $\beta_{com} = 2.8 \cdot 10^{-9}\, s^{-1}$, $1/\mu = 2$ days, $1/\gamma = 4$ days, $p_A = 1/3$.

Closure strategy (threshold, duration)	Targeted class	Targeted grade	Whole school
No closure	34.1	34.1	34.1
3, 24 h	29.4	28.2	27.7
3, 48 h	25.3	24.2	21.9
3, 72 h	22.9	21.3	18.6
3, 96 h	21.4	18.9	15.6
3, 120 h	20.3	17.1	12.0
3, 144 h	19.9	16.8	7.3
2, 24 h	26.7	26.7	25.6
2, 48 h	21.6	21.3	20.4
2, 72 h	18.1	17.3	15.5
2, 96 h	16.2	13.7	12.2
2, 120 h	10.2	9.0	5.6
2, 144 h	9.4	7.4	3.3

TABLE VIII. Percentage of realizations leading to an attack rate higher than 10%, for the various mitigation strategies with various thresholds and closure durations. Parameter values (durations distributed according to Weibull distributions of shape parameter 4): $\beta = 3.5 \cdot 10^{-4}\, s^{-1}$, $\beta_{com} = 2.8 \cdot 10^{-9}\, s^{-1}$, $1/\mu = 2$ days, $1/\gamma = 4$ days, $p_A = 1/3$.

Closure strategy (threshold, duration)	Targeted class	Targeted grade	Whole school
3, 24 h	162 [98,193]	166 [109,196]	174 [143,200]
3, 48 h	129 [35,178]	137 [35,187]	166 [77,197]
3, 72 h	113 [32,169]	116 [31,181]	155 [34,197]
3, 96 h	97 [27,154]	98 [28,168]	137 [27,196]
3, 120 h	85 [29,148]	83 [27,161]	120 [27,196]
3, 144 h	76 [29,142]	66 [27,142]	91 [25,192]
2, 24 h	164 [96,196]	166 [70,201]	171 [117,202]
2, 48 h	129 [40,184]	138 [38,187]	174 [148,199]
2, 72 h	101 [28,170]	122 [30,182]	170 [136,195]
2, 96 h	89 [29,160]	114 [30,181]	148 [33,192]
2, 120 h	85 [27,163]	85 [27,175]	140 [29,193]
2, 144 h	64 [27,146]	72 [27,176]	138 [29,189]

TABLE IX. Average final number of cases, computed for the realisations leading to an attack rate larger than 10%, for the various mitigation strategies; the brackets provide the 5th and 95th percentiles. The baseline case given by the simple isolation of symptomatic children is indicated as "No closure". Parameter values (durations distributed according to Weibull distributions of shape parameter 3): $\beta = 3.5 \cdot 10^{-4} s^{-1}$, $\beta_{com} = 2.8 \cdot 10^{-9} s^{-1}$, $1/\mu = 2$ days, $1/\gamma = 4$ days, $p_A = 1/3$.

Closure strategy (threshold, duration)	Targeted class	Targeted grade	Whole school
No closure	176 [132,200]	176 [132,200]	176 [132,200]
3, 24 h	162 [73,192]	167 [90,197]	173 [112,199]
3, 48 h	136 [47,182]	141 [37,188]	167 [44,199]
3, 72 h	108 [32,167]	112 [30,178]	156 [33,198]
3, 96 h	98 [29,162]	97 [29,170]	137 [29,194]
3, 120 h	85 [29,152]	79 [27,156]	106 [27,190]
3, 144 h	76 [28,139]	62 [26,142]	73 [25,187]
2, 24 h	163 [64,196]	166 [107,199]	171 [80,202]
2, 48 h	128 [32,186]	143 [37,193]	172 [138,199]
2, 72 h	103 [30,171]	117 [29,185]	165 [141,199]
2, 96 h	83 [28,161]	98 [27,178]	154 [33,201]
2, 120 h	81 [27,170]	75 [26,154]	132 [33,194]
2, 144 h	56 [26,131]	61 [26,152]	98 [41,199]

TABLE X. Average final number of cases, computed for the realisations leading to an attack rate larger than 10%, for the various mitigation strategies; the brackets provide the 5th and 95th percentiles. The baseline case given by the simple isolation of symptomatic children is indicated as "No closure". Parameter values (durations distributed according to Weibull distributions of shape parameter 4): $\beta = 3.5 \cdot 10^{-4} s^{-1}$, $\beta_{com} = 2.8 \cdot 10^{-9} s^{-1}$, $1/\mu = 2$ days, $1/\gamma = 4$ days, $p_A = 1/3$.

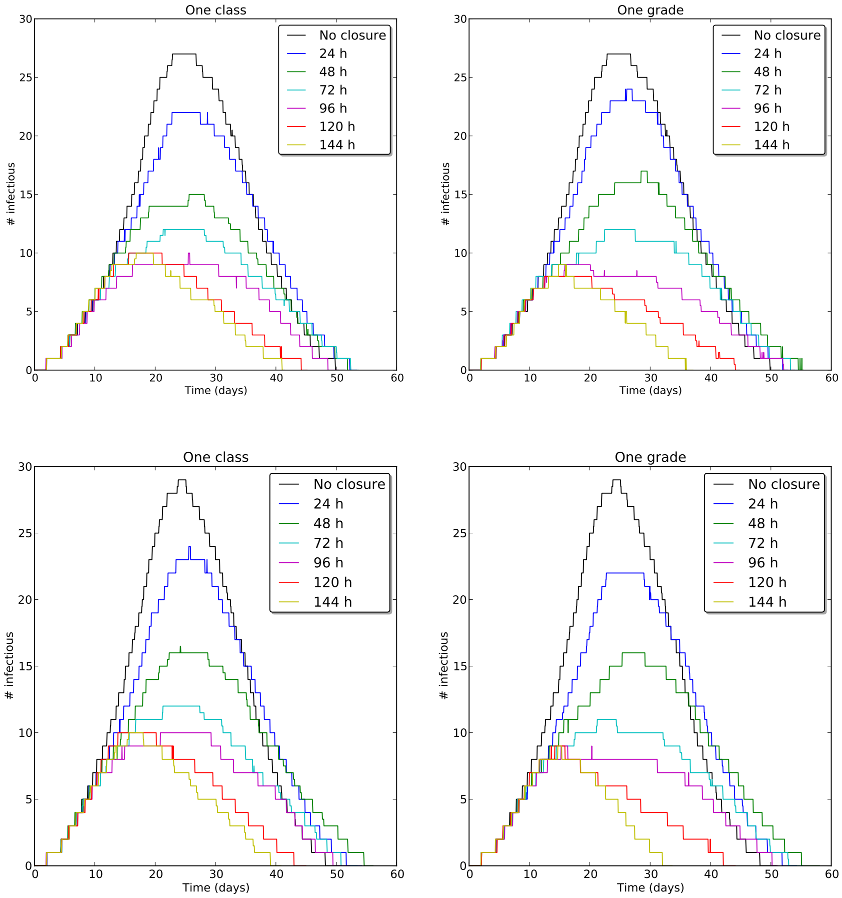

FIG. 4. Temporal evolution of the median number of infectious individuals for several closure durations, at a fixed closure-triggering threshold of 3 symptomatic cases. Left: targeted class closure. Right: targeted grade closure. Only runs with an attack rate (AR) higher than 10% are taken into account. Parameter values: $\beta = 3.5 \cdot 10^{-4}\, s^{-1}$, $\beta_{com} = 2.8 \cdot 10^{-9}\, s^{-1}$, $1/\mu = 2$ days, $1/\gamma = 4$ days, $p_A = 1/3$. Latent and infectious period durations are distributed according to Weibull distributions of shape parameters 3 (top) and 4 (bottom).

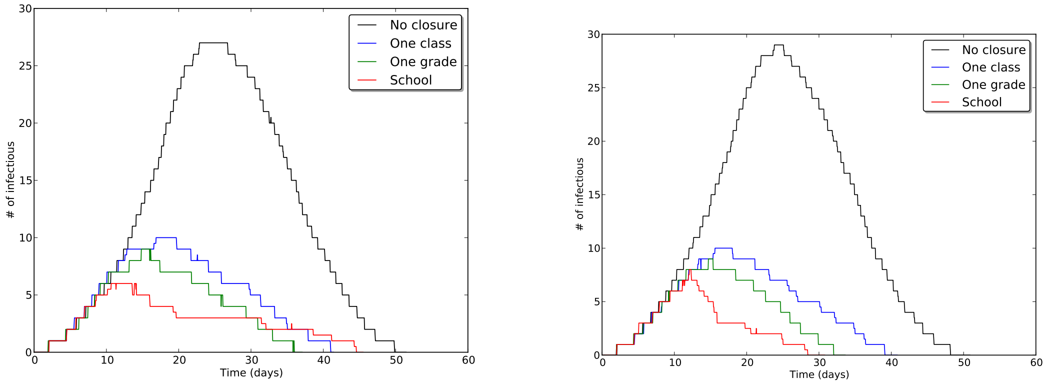

FIG. 5. Temporal evolution of the median number of infectious individuals for the targeted class, targeted grade, and whole school closure strategies, at a fixed closure-triggering threshold of 3 infectious individuals and closure duration of 144 hours (6 days). The no-closure scenario is provided for reference. Only realizations with an attack rate (AR) higher than 10% are taken into account. Parameter values: $\beta = 3.5 \cdot 10^{-4}\, s^{-1}$, $\beta_{com} = 1.4 \cdot 10^{-9}\, s^{-1}$, $1/\mu = 2$ days, $1/\gamma = 4$ days, $p_A = 1/3$. Latent and infectious period durations are distributed according to Weibull distributions of shape parameters 3 (left) and 4 (right).

COMPARISON OF TARGETED AND RANDOM CLASS AND GRADE CLOSURES

We consider here strategies based on random closure of classes: whenever the number of symptomatic infectious individuals detected in any class reaches a certain threshold,

(iv) one random class, different from the one in which symptomatic individuals were detected, is closed ("random class closure" strategy)

(v) this class and a randomly chosen one in a different grade are closed ("mixed class closure" strategy).

We use here $\beta = 6.9 \cdot 10^{-4} s^{-1}$, $\beta_{com} = 2.8 \cdot 10^{-9} s^{-1}$, $1/\mu = 1$ day, $1/\gamma = 2$ days, $p_A = 1/3$.

Tables XI and XII compare the effect of the targeted class and grade closure with the partially random closure of one or two classes. Closing one class chosen at random (different than the one in which the infectious individuals are detected) leads only to a marginal decrease in the probability to obtain an attack rate higher than 10% and in the number of individuals affected by large spreads.

In the mixed strategy on the other hand, the class in which the infectious individuals have been detected is closed, and a second one, chosen at random in a different grade, is closed as well. This leads to an effect almost as strong as the targeted grade closure strategy.

Figures 6 and 7 report the temporal evolution of the median number of infectious individuals in the targeted and random strategies, leading to the same conclusion: the targeted closure of a class leads to a much smaller peak than the closure of a random class; the mixed strategy leads on the other hand to an epidemic curve that is very close to the case of a targeted grade closure.

Closure strategy (Threshold, duration)	Targeted class	Random class	Targeted grade	Mixed
No closure	26.4	26.4	26.4	26.4
3, 24 h	22.8	26.2	21.8	22.1
3, 48 h	21.8	24.4	17.0	18.1
3, 72 h	18.6	26.0	15.8	17.6
3, 96 h	16.6	23.2	14.1	16.5
2, 24 h	22.2	23.8	21.5	22.2
2, 48 h	16.8	24.3	17.0	17.9
2, 72 h	11.9	24.8	11.2	14.4
2, 96 h	10.2	23.8	8.7	13.1

TABLE XI. Percentage of runs leading to an attack rate larger than 10% for the targeted and random closure strategies. $\beta = 6.9 \cdot 10^{-4} s^{-1}$, $\beta_{com} = 2.8 \cdot 10^{-9} s^{-1}$, $1/\mu = 1$ day, $1/\gamma = 2$ days, $p_A = 1/3$.

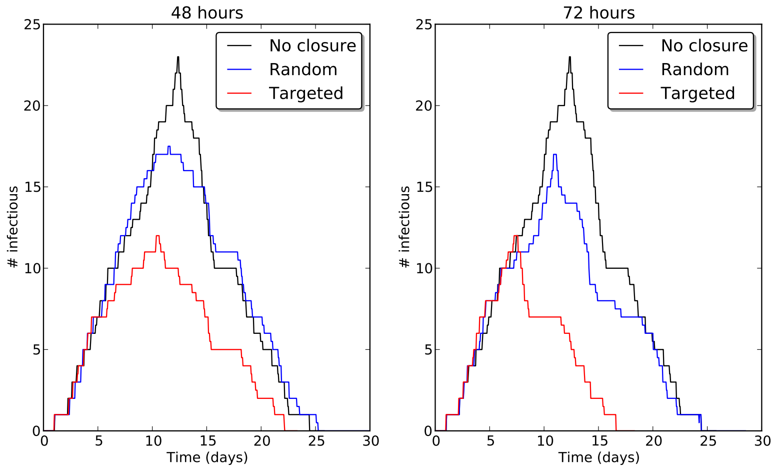

FIG. 6. Temporal evolution of the number of infectious individuals for targeted and random class closure strategies, for a closure-triggering threshold of 3 infectious individuals, compared with the scenario in which no closure is implemented. The duration of the class closures is 48 hours (Left) and 72 hours (right). $\beta = 6.9 \cdot 10^{-4} s^{-1}$, $\beta_{com} = 2.8 \cdot 10^{-9} s^{-1}$, $1/\mu = 1$ day, $1/\gamma = 2$ days, $p_A = 1/3$. Only realizations with attack rate (AR) larger than 10% are taken into account.

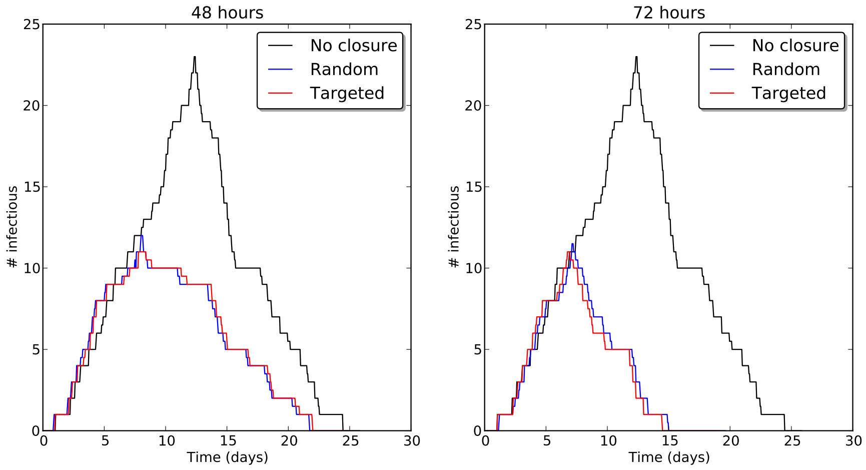

FIG. 7. Temporal evolution of the number of infectious individuals for the targeted grade closure and the mixed closure strategies, for a closure-triggering threshold of 3 infectious individuals, compared with the scenario in which no closure is implemented. The duration of the class closures is 48 hours (Left) and 72 hours (right). In the mixed closure strategy, the class in which the infectious individuals are detected is closed, as well as a second class from a different grade. $\beta = 6.9 \cdot 10^{-4} s^{-1}$, $\beta_{com} = 2.8 \cdot 10^{-9} s^{-1}$, $1/\mu = 1$ day, $1/\gamma = 2$ days, $p_A = 1/3$. Only realizations with attack rate (AR) larger than 10% are taken into account.

Closure strategy (Threshold, duration)	Targeted class	Random class	Targeted grade	Mixed
No closure	154 [106,209]	154 [106,209]	154 [106,209]	154 [106,209]
3, 24 h	133 [90,194]	151 [106,207]	131 [90,192]	132 [90,195]
3, 48 h	100 [68,175]	145 [103,201]	99 [68,174]	102 [70,176]
3, 72 h	71 [39,135]	130 [89,193]	63 [37,133]	64 [37,135]
3, 96 h	66 [38,124]	123 [87,191]	58 [37,122]	63 [37,133]
2, 24 h	130 [88,192]	153 [105,207]	126 [83,189]	128 [84,192]
2, 48 h	94 [67,169]	150 [104,207]	91 [66,169]	95 [67,170]
2, 72 h	54 [36,120]	135 [93,190]	52 [36,119]	56 [37,123]
2, 96 h	51 [37,109]	119 [84,189]	45 [35,104]	48 [35,106]

TABLE XII. Average final number of cases of the spread, for realisations with $AR > 10\%$, for the targeted and random strategies. $\beta = 6.9 \cdot 10^{-4} s^{-1}$, $\beta_{com} = 2.8 \cdot 10^{-9} s^{-1}$, $1/\mu = 1$ day, $1/\gamma = 2$ days, $p_A = 1/3$. The brackets give the 5th and 95th percentiles.
