# Supplementary figures and images for: Mitigation of infectious disease at school: targeted class closure vs school closure
Source: BMC Infect Dis. 2014 Dec 31;14:695. doi: 10.1186/s12879-014-0695-9 (PMC4297433; doi:10.1186/s12879-014-0695-9)

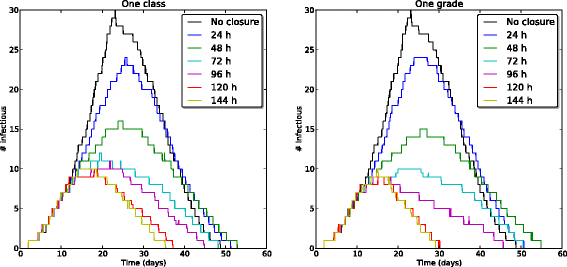

Supplement: Supplementary file 3 — Authors’ original file for figure 1 [file 12879_2014_695_MOESM3_ESM.gif]

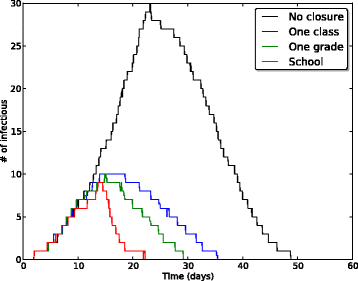

Supplement: Supplementary file 4 — Authors’ original file for figure 2 [file 12879_2014_695_MOESM4_ESM.gif]

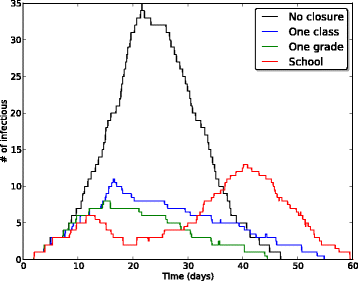

Supplement: Supplementary file 5 — Authors’ original file for figure 3 [file 12879_2014_695_MOESM5_ESM.gif]
